# An empirically grounded conceptual framework of the determinants of economic resilience: Insights from seven major Canadian regions

**DOI:** 10.1177/0308518X251391204

**Published:** 2025-11-19

**Authors:** Jesse Sutton, Godwin Arku

**Affiliations:** 1 Department of Geography and Environment, Social Science Centre, Western University, London, ON, Canada

**Keywords:** determinants, resilience, regions, Canada, interviews, conceptual framework

## Abstract

Investigating the determinants of resilience has been a core research agenda in the regional economic resilience literature. However, no comprehensive conceptual framework of the determinants of resilience currently exists. Instead, the determinants are typically presented as a set or list of factors that influence the economic resilience of regions. A more comprehensive conceptual framework, illustrating how such determinants interact, is therefore needed. To address this gap, this paper develops an empirically grounded conceptual framework of the determinants of regional economic resilience. To do so, this paper conducted in-depth interviews with economic development practitioners (*n* = 41) from seven major Canadian regions. In the developed conceptual framework, the paper highlights the interactive nature of the determinants of resilience, the importance of recognizing the ecological limits of regions, and the roles that firm-based and system-based actors play in shaping regional economic resilience.

## Introduction

Identifying the determinants of resilience has been one of the main research agendas in the resilience literature since the notion of resilience was incorporated into economic geography and regional studies in the late 2000s. The determinants of resilience refer to the myriad of factors, underscored by endogenous and exogenous dynamics, that jointly shape the economic resilience of regions (Hu and Hassink, 2020; Martin and Sunley, 2015). Currently, the determinants are typically presented as a list or a set of factors that influence the economic resilience of regions (Crescenzi et al., 2016; Evenhuis, 2017; Giannakis and Bruggeman, 2017; Sutton et al., 2023), with some scholars broadly noting that such factors interact (Hu and Hassink, 2020; Martin and Sunley, 2015). However, such overviews provide a limited understanding of the determinants of resilience and their complexities, hindering the development of a greater understanding of regional economic resilience and reducing the ability of such literature to support policymakers’ and local officials’ resilience-building efforts. A conceptual framework of the determinants of resilience, moving past long lists or sets of factors that influence regional economic resilience and highlighting how such factors interact, is therefore needed.

To address this gap, the present paper offers an empirically grounded conceptual framework of the determinants of resilience. In doing so, it answers the research question: how do the determinants of resilience interact? Overall, the paper provides a novel contribution to the resilience literature, expanding our current understanding of the dynamic and complex nature of the factors that influence the economic resilience of regions and how such factors interact.

To provide such a conceptual framework, this paper conducted in-depth interviews with 41 economic development practitioners (EDPs) from 7 major Canadian regions. Specifically, the experiences and perceptions of EDPs were investigated. Unlike firms, business associations, universities, community organizations, and other actors who operate in select domains within an economy, EDPs are one of the only actors who are highly intertwined in almost every aspect of an economy (Sutton et al., 2024b), as they are specifically tasked with managing the overall economic and social well-being of their localities (Arku, 2015; Leigh and Blakely, 2017). As a result, EDPs are ideal candidates to gain an in-depth understanding of issues related to regional economic resilience and other regional phenomena.

Such qualitative approaches are important as they provide valuable insight that quantitative methods cannot glean (Gong and Hassink, 2020). In fact, scholars in the resilience literature have long advocated for qualitative approaches to be employed as such approaches are able to provide deeper and more nuanced insight (Bristow and Healy, 2014; Cowell, 2013; Evenhuis, 2017; Hill et al., 2008). Indeed, the level of insight provided by qualitative approaches, particularly using in-depth interviews, makes them well-suited to achieve the research objective of this study, which is to develop a conceptual framework of the determinants of resilience.

Furthermore, Canada provides an informative case study as the country, like others, has been hit by many shocks (e.g. tariff and trade disputes, natural disasters, recessions, pandemics, major industrial plant closures, and so on) since the beginning of the 21st century, resulting in the notion of resilience and resilience-building efforts being at the forefront of many economic development departments across the nation. In other words, EDPs in Canada are well-informed about resilience and are actively engaged in resilience-building activities (Sutton et al., 2024a). Also, the empirically grounded conceptual framework is widely applicable since Canadian regional economies operate similarly to those in other Western countries, such as the United States of America, Australia, and European countries. As a result, the developed framework can support policymakers’ and local officials’ resilience-building efforts in regions in Canada and abroad.

The paper is structured as follows. The next section provides an overview of regional economic resilience and the current conceptualization of the determinants of resilience. The following section details the methods employed. The penultimate section presents the empirically grounded conceptual framework. The last section concludes with a brief discussion.

## Background

Since the 2008 Great Recession, the notion of economic resilience has gained considerable attention in economic geography and regional studies. It is generally defined as “*the ability of regional economies to resist and adapt to or transform in the face of shocks and subsequently recover to maintain or improve their pre-shock economic performance*” (Sutton et al., 2023: 6, original emphasis). The notion of economic resilience is entrenched in evolutionary thinking due to insights gained from evolutionary economic geography. Scholars, especially evolutionary economic geographers, argue that economies are marked by relentless and constant change, rejecting equilibrist notions from traditional economics (Christopherson et al., 2010; Dawley et al., 2010; Hassink, 2010; Simmie and Martin, 2010) that cannot adequately explain the heterogeneous resilience performance among regions (Pike et al., 2010). Indeed, economies do not reach or tend towards a steady or equilibrium state as they experience continuous incremental change, arising from their encompassing actors’ adaptive actions and behaviours (Boschma and Frenken, 2006; Martin, 2010).

Overall, economic resilience is considered a dynamic process (Martin and Sunley, 2015; Sutton et al., 2023), consisting of five interrelated dimensions: *preparation* (i.e. the preparedness of regions for shocks), *vulnerability* (i.e. the susceptibility of regions to shocks), *resistance* (i.e. the sensitivity of regions to shocks once impacted), *response* (i.e. the degree of adaptive behaviour exerted by regions in the face of shocks), and *recoverability* (i.e. the extent to which regions recover from shocks). The progression of regions through these dimensions ultimately determines their resilience performance. However, the response dimension is the most significant as it reflects the degree to which regionally operating actors adapt to shock (Sutton et al., 2023). Furthermore, the dynamic process of resilience is a recursive one in which the resilience behaviour of regions (e.g. changes in regional structure and functions) during shocks influences their post-crisis development and, hence, resilience to future shocks (Martin and Sunley, 2015, 2020; Simmie and Martin, 2010).

The majority of conceptual development in the resilience literature has focused on the process of resilience (e.g. Boschma, 2015; Evenhuis, 2017; Hu and Hassink, 2020; Martin, 2012; Martin and Sunley, 2015; Pendall et al., 2010; Simmie and Martin, 2010; Sutton et al., 2023; Trippl et al., 2024). As a result, economic resilience has evolved into a well-developed concept due to continual conceptual advancements (Sutton et al., 2023).^1^ In contrast, the determinants of resilience have received little conceptual development, primarily being the subject of empirical investigation (e.g. Doran and Fingleton, 2018; Ray et al., 2017; Wang and Wei, 2021). In investigating the determinants of resilience, scholars have identified a handful of factors that influence regional economic resilience.

However, scholars largely focus on the structural factors that shape the economic resilience of regions (Bristow and Healy, 2014; Martin and Sunley, 2020), such as industrial structure, business characteristics, supply chains, and sectoral composition. By far, the factor that has received the most attention is industrial structure, focusing on the influence of industrial diversity. Scholars emphasize that regions with a diversified industrial structure are more resilient to shocks than those with a specialized structure (Martin and Sunley, 2015). Furthermore, the type of variety, either related or unrelated, that comprises a diversified industrial structure is also suggested to positively affect the economic resilience of regions, yet in different ways (Boschma, 2015; Frenken et al., 2007). Related variety refers to a variety of industries that share similar competencies, whereas unrelated variety refers to a variety of industries that do not (Boschma and Iammarino, 2009; Hartog et al., 2012).^2^

The primary focus on structural factors has led scholars to criticize the resilience literature for neglecting the role of actors (Bristow and Healy, 2014, 2020; Kurikka and Grillitsch, 2021; Lemke et al., 2023). Currently, the range of actors that influence regional economic resilience and how they do so is underexplored, which is surprising given that actors are noted to be at the core of the evolutionary resilience process (Martin and Sunley, 2015; Sutton et al., 2023). However, some progress has been made, with recent research examining the role of policymakers (Bristow and Healy, 2018) and economic development practitioners (Sutton et al., 2024a). Overall, despite the empirical attention given to the determinants of resilience, they still remain a black box in the resilience literature, requiring further exploration (Hu and Hassink, 2020).

Aside from empirical investigations, discussions on the determinants of resilience have largely resided with scholars providing lists or outlining sets of factors that influence regional economic resilience. For example, Boschma (2015) outlines how industrial, network, and institutional structures influence regional economic resilience. Crescenzi et al. (2016) highlight how regional economic and labour market performance during the 2008 Great Recession is influenced by three main sets of pre-crisis factors: macroeconomic factors, regional industrial-mix, and regional competitiveness. Giannakis and Bruggeman (2017) present 10 regional factors that influence regional labour market performance during recessions. Evenhuis (2020) provides a thorough list of seven domains in regional economies that shape regional adaptation and resilience: economic base, technology and knowledge base, capital and finance, labour market and skills, built environment, institutional arrangements, and policy. Sutton et al. (2023) present a list of 14 determinants identified in the resilience literature.

However, scholars (Hu and Hassink, 2020; Martin and Sunley, 2015, 2020) are starting to move past simply outlining the determinants of resilience by noting their interactive nature. For example, Martin and Sunley (2020) note that regional economic resilience is “determined by the dynamics of five main economic, interacting subsystems: the structural and business subsystem; the labour market subsystem; the financial subsystem; the governance subsystem; and the character of local individual agency and decision-making” (p. 25). Simply put, scholars emphasize that the factors that influence the economic resilience of regions do not operate in isolation (Hu and Hassink, 2020). Despite this recognition, the interactive nature of the determinants of resilience has received little attention overall. Indeed, further investigation into how precisely the determinants interact is required, moving past broad and ambiguous statements.

## Methods

To develop an empirically grounded conceptual framework of the determinants of resilience, illustrating how such determinants interact, in-depth interviews were relied upon. Such a qualitative approach is well-established for examining various phenomena surrounding economies (Arku, 2014; Rubin, 1988). Using this approach allows one to gain valuable insights into the determinants of resilience that are typically challenging to tease out, especially when using quantitative approaches.

Precisely, in-depth interviews with 41 EDPs (i.e. economic development directors, managers, senior staff, and so on) from 7 major Canadian regions (i.e. Toronto, Montreal, Vancouver, Calgary, Edmonton, Ottawa-Gatineau, and Halifax regions) were conducted in 2024 (Table 1). The regions of Toronto, Ottawa-Gatineau, and Montreal are located in Central Canada (i.e. in the provinces of Ontario and Quebec), with the Greater Toronto Area containing the City of Toronto, the largest city in the country. The greater areas of Calgary and Edmonton are situated in Western Canada, specifically in the province of Alberta, while the greater area of Vancouver also lies within Western Canada but in the province of British Columbia. The latter three urban areas are rapidly expanding regions, with Calgary and Edmonton experiencing the greatest population growth in Canada over the past two decades. Finally, the greater area of Halifax is located in Atlantic Canada, specifically in the province of Nova Scotia, and is one of Canada’s oldest urban regions. The first six regions (i.e. Toronto, Montreal, Vancouver, Ottawa-Gatineau, Calgary, and Edmonton) are Canada’s most populated urban areas (Bone, 2022). Halifax was included to gain insight from Atlantic Canada. These regions were selected to provide a diverse and holistic perspective by encompassing a range of locations, spanning the east-west axis that underscores Canada’s economic geography and economic development experiences.

**Table 1.** Overview of Canadian regions.

Regions	Municipalities included	Population 2023 (growth 2001–2023)
Toronto	Toronto; Mississauga; Brampton; Markham; Vaughan; Aurora; York; Durham; Oshawa; and Whitby	7.3 million people (40%)
Montreal	Montreal; Laval; Longueil; Terrebonne; Saint-Jean-sur-Richelieu; and Dorval	4.5 million people (24%)
Vancouver	Vancouver; Surrey; Richmond; Coquitlam; North Vancouver; New Westminster; and Port Moody	3.0 million people (43%)
Calgary	Calgary; Airdrie; Cochrane; Chestermere; Canmore; and Okotoks	1.7 million people (72%)
Edmonton	Edmonton; Strathcona County; Sturgeon County; Fort Saskatchewan; Leduc County; and Lamont County	1.6 million people (62%)
Ottawa-Gatineau	Ottawa; Gatineau; Stormont, Dundas and Glengarry; Leeds and Grenville; and Renfrew County	1.6 million people (38%)
Halifax	Halifax Regional Municipality	519,000 people (32%)

Source of Population Stats: Statistics Canada (2024).

*Note.* Population Statistics based on Census Metropolitan Areas population estimates.

### Study participants

EDPs were selected to be interviewed due to their economic development experience, intimate knowledge of their economy and surrounding economies, and their responsibilities to support and manage the economic well-being of their locality. The EDPs’ experience in the field of economic development ranged from 4 months to 26 years, with an average of 11 years. EDPs worked in economic development positions within their municipality. A minimum of five EDPs per region were targeted.^3^ This selection strategy was undertaken to gain an exhaustive sample pool in which comprehensive insights on the determinants of regional economic resilience could be gleaned.

EDPs were contacted by their publicly available professional email addresses. More than 60 email invitations were sent out over roughly a 6-month period in 2024, of which 41 EDPs agreed to and participated in the study. Approximately six to eight interviews were conducted in each region. However, 10 interviews were completed in the Greater Toronto Area, due to (1) a high volume of positive responses to participate in the research study and (2) the region having the most municipalities with economic development offices in Canada. Furthermore, only one interview was conducted in the greater area of Halifax as the region only contains one municipality (i.e. the Halifax regional municipality).

### Data collection and analysis

The interviews were semi-structured, with EDPs being asked open-ended questions. The duration of the interviews was on average 64 minutes, ranging from 24 to 114 minutes in length. To analyze the data, a grounded and inductive approach was used. Specifically, the analysis was guided by the evaluation criteria of credibility, transferability, dependability, and confirmability to ensure qualitative rigour. That is, the criteria enabled us to illustrate the significance of a single case (credibility) and move beyond it (transferability) with a high degree of confidence (dependability and confirmability; Baxter and Eyles, 1997). Aligning with the research objective of this paper, the purpose of the interviews was to develop a conceptual framework of the determinants of resilience, illustrating how such determinants interact. The following is a sample of interview questions: What are the underlying features that make economies resilient? How do these features influence economies’ resilience? Based on your knowledge of past local shocks, how did your economy react and respond to such disruptions? Who are the key agents enabling economies to adapt and respond to shocks? How do the determinants of resilience interact?

Interviews were conducted over Zoom by two researchers and audio recorded. Interviews with EDPs from the province of Quebec were conducted in French, as French is the official language of that province. A French interviewer was hired to conduct and transcribe verbatim the interviews in French. NVivo software was used to transcribe the interviews verbatim and thematically analyze the transcripts, a process guided by the research objective. Thematic analysis is useful for identifying, analyzing, and interpreting qualitative data (Clarke and Braun, 2017). Particularly, the thematic analysis was undertaken by two researchers. Thematic codes were derived from the transcripts, which were vetted line-by-line. This coding process is the most rigorous, enabling a generative and exhaustive analysis (Clifford and Valentine, 2003; Corbin and Strauss, 2014). Both the transcripts and the thematic codes were double-checked to ensure accuracy. In recent years, such qualitative approaches have been increasingly employed in economic geography and regional studies (Arku, 2014; Rubin, 1988; Sutton et al., 2022a, 2022b) to glean insight from the perspectives and experiences of local officials.

As noted, an inductive approach was used for the coding process, with all codes arising organically from the interviews (Clarke and Braun, 2017). First, an initial round of open coding was conducted to capture all explicit and implicit references to the determinants of resilience and their interaction. As codes emerged, the list of codes was iteratively revised, with codes being consolidated, refined, or split for thematic fit and clarity (Corbin and Strauss, 2014; Saldaña, 2021). Once the initial set of codes was established, axial coding was conducted. That is, the open codes were reorganized into categories and subcategories based on their relationship and similarities (Corbin and Strauss, 2014), as indicated by the interviewees. Axial coding resulted in the emergence of themes (e.g. actors) and sub-themes (e.g. firm-based and system-based actors). From the themes and sub-themes, a final conceptual framework was developed, which illustrates the interactive nature of the determinants of resilience.

Given the nature of our research method, excerpts are used to illustrate the findings. The abbreviation “P” is used to denote participants. In addition, the interviewees were randomly assigned a numerical code, ranging from 1 to 41, throughout the results section.

## Results

**Figure 1.**
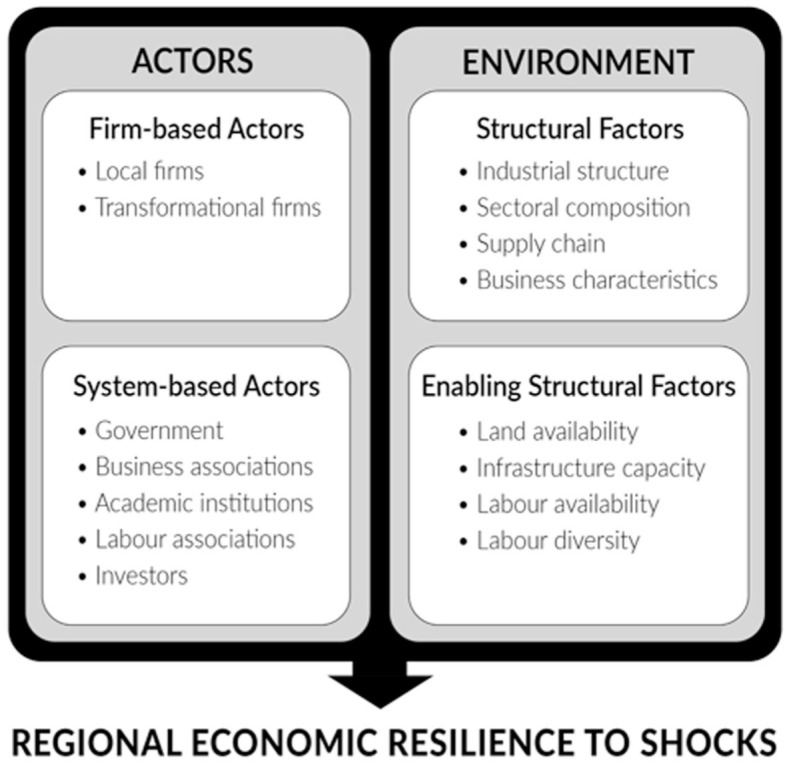
The determinants of regional economic resilience.

The findings from the thematic analysis provided a comprehensive conceptual framework of the determinants of resilience, demonstrating how such determinants interact. The overarching finding from the in-depth interviews is that there are two main categories of determinants: (1) actors and (2) environmental factors (Figure 1). Actors were noted to be the determinants in regional economies that exhibit agency. Such actors refer to those who operate within the region, such as local firms, multi-scale institutional actors, or transnational corporations. Environmental factors were highlighted as the determinants that reflect the structural composition of a regional economy.

Stemming from the results, both actors and environmental factors can be further dichotomized into subcategories. Regarding actors, they can be further split into (1) firm-based actors who engage in economic activity and (2) system-based actors who support the behaviour and actions of firm-based actors. One interviewee from the Calgary region, highlighting the dichotomy between actors, noted, “when you talk about resiliency, it’s really about the people who build businesses and not really about the municipality or the people trying to help them out” (P21). The dichotomy illustrated by the previous quote was echoed by many other interviewees, overall stressing that some actors engage in economic activity and others support such actors. In this view, firm-based actors directly affect regional economic resilience, whereas system-based actors have an indirect influence.

Similarly, environmental factors arising from the interviews can be divided into (1) structural factors that influence the impact of shocks and/or regions’ adaptive capacity and (2) enabling structural factors that allow structural factors to be developed, and adaptive behaviour to unfold. Illustrating this dichotomy, a diversified industrial structure (i.e. a structural factor) was commonly noted to enhance the economic resilience of regions. This point was emphasized by one interviewee from the Edmonton region who simply noted, “the diversity of our industries helps us to be more resilient” (P40). However, interviewees stressed that enabling structural factors, such as available employment land, were needed for diversification to occur, as expressed by one interviewee from the Ottawa-Gatineau region who stated, “So there’s not a lot of space in our region anymore to welcome new firms or diversify our economy” (P13). The dichotomy of environmental factors arose throughout the results in various forms. In this vein, structural factors directly influence regional economic resilience, whereas enabling structural factors have an indirect effect.

Notably, the findings indicate that the determinants of resilience interact in a highly complex and dynamic manner. Precisely, the interaction between firm-based actors and the other determinants ultimately governs regions’ response to crises and their structural evolution during but primarily following crises. Focusing first on the former, firm-based actors’ behaviour during shocks is aided, to varying degrees, by system-based actors and influenced by the opportunities brought by structural factors to recombine knowledge and innovate. In response to shocks, firm-based actors either build on their current infrastructure and capabilities or expand into new or different ventures made available by enabling structural factors. Simply put, the ability of firm-based actors to respond is shaped by system-based actors, structural factors, and enabling structural factors that collectively comprise a regional economy during a crisis. Concentrating next on the latter, structural factors are largely shaped pre- and post-crisis by firm-based actors engaging with enabling structural factors, the extent to which is determined by system-based actors. In other words, the influence that structural factors have mainly results from firm-based actors, system-based actors, and enabling structural factors that comprise a regional economy pre-crisis. The remaining subsections will provide an overview of the determinants of resilience and illustrate how they interact in more depth.

### Firm-based actors

Importantly, interviewees stressed that firm-based actors (i.e. local and external firms) directly shape the economic resilience of regions. When asked who the key agents are enabling economies to adapt and respond to shocks, interviewees answered that they are market actors, referring to firms that engage in economic activity. Expressing this point, one interviewee from the Montreal region answered: “it’s the private business owners that really make it run. They’re the agents of it all . . .. It’s the resiliency that comes from private businesses” (P29). The underlying reason why interviewees noted firms were central to the economic resilience process was that such actors are strongly tied to the market, unlike the public sector, and their adaptive actions and behaviour during crises drastically impact the well-being of economies, such as by providing employment. This point was reflected by an interviewee from the Halifax region who stated, “if businesses are able to kind of adapt or they’re able to transform or kind of shift operations in any meaningful way, then that certainly creates resilience because then they’re not laying off massive amounts of employees” (P41). Conversely, interviewees noted, which will be illustrated in the following subsection, that other actors typically support firm-based actors’ capacity to respond to shocks.

Interestingly, interviewees highlighted that firm-based actors’ influence depends on their adaptive behaviour, as not all firms adapt, or are able to, during shocks. Indeed, some firm-based actors simply act in a reactive (or passive) manner, while others are proactive, taking shocks as opportunities. As expressed by one interviewee (P22) from the Vancouver region, some firm-based actors:

try to absorb shocks. They’re not quite resilient enough to bounce back on their own . . . the majority of our businesses are typically like that. Then, you have those that will quickly adapt . . . those, I refer to those as your innovators, your entrepreneurs, those that are like serial entrepreneurs that just continue to be able to resist that type of change, or quickly move and adapt, . . . and it is those businesses who really support our region’s resiliency.

Providing an illustrative example, an interviewee (P07) from the Toronto region stated:

There was a guy [in our region] that adapted his rubber company to make face shields for the health sciences sector early on in the [COVID-19] pandemic when nobody was buying rubber products anymore. He also made those plexiglass barriers that go in front of a kiosk or in front of your cash register or between booths in a restaurant. So, they were like, oh, we’ve got plexiglass. We know how to form stuff. Let’s make those things. It’s those kinds of people that support regions’ [economic] resilience the most, their reaction is more proactive and their reaction is not what everybody else would do, which is, oh, well, I need to start planning for layoffs. I need to start planning for decreased revenue. I need to notify all of our regular buyers that we’re still here, but we’re probably going to be ramping down. He’s the kind of guy that’s like, we need new suppliers.

The previous example was one of many given during the interview process in which interviewees indicated that regional economic resilience stems largely from firm-based actors’ adaptive behaviour and actions. However, as illustrated below, other important actors and factors also influence the economic resilience of regions as well as shape firm-based actors’ adaptive capacity.

### System-based actors

Despite system-based actors playing an indirect role in regional economic resilience, they were still seen as key actors. This point was expressed by one interviewee (P25) from the Vancouver region, who stated:

ultimately [regional economic] resilience relies on the businesses themselves wanting to invest, wanting to grow, to adapt, but the role that governments, not-for-profits, partner organizations play is extremely important to support businesses. So, they provide great grant programs, great funding programs, export development programs, all of which can play an important part in helping businesses adapt.

Indeed, interviewees highlighted that system-based actors support firm-based actors’ capacity to respond to shocks. Illustrating this point, an interviewee from the Montreal region commented, “it’s more and more important for communities to have people who work at different levels of economic planning, to ensure adaptation. Without this, businesses would have a lot more difficulty adapting” (P31).

Furthermore, interviewees stressed that it is the collective efforts of system-based actors at various scales that shape the capacity of firm-based actors to respond to shocks. In this manner, system-based actors operating in regional economies are more than the sum of their parts. Reflecting this sentiment, one interviewee (P12) from the Ottawa-Gatineau region, when asked who the key agents are during shocks, remarked:

the people, whose work is to support businesses, are there to help at their respective levels. Sometimes, that takes the form of loans, help with restructuring, help with relocating . . . Businesses have needs at a lot of different levels and each of us, working at our different levels, are key agents. It is the amalgamation of these services that makes us capable of supporting a business.

Or, as expressed by an interviewee (P29) from the Montreal region:

the main leaders in supporting the resiliency of Montreal is a collective. It has to be a collective of the region leaders, provincial leaders, and then our local business leaders and the municipality . . . Not having that group would have been pretty detrimental to having our businesses bounce back after a COVID event.

Moreover, system-based actors were noted to primarily support firm-based actors’ adaptive behaviour. However, in the case of government, it was stated also to have the potential to hinder firm-based actors’ adaptive capacity. Expressing this point, an interviewee from the Calgary region simply noted, “government very unintentionally provides a lot of barriers for businesses” (P17). For example, an interviewee from the Vancouver region reported that manufacturers in Vancouver during the COVID-19 pandemic were “pushing the government to reduce the red tape [around getting permits to change their machinery], as they were trying to convert their, or change their machinery to build COVID-related things. And the government did not respond too well to it. It took a long time” (P24). The interviewee went on to note that “it was just a nightmare, especially for the film industry. So, they tried to keep things going and have lots of testing. And in order to do quick testing, you had to create different machines, and to have permits for those machines that took a long time.” These quotes highlight the potential dual influence of system-based actors, both hindering and supporting firm-based actors.

Overall, interviewees emphasized the importance of five main system-based actors: (1) government, (2) business associations, (3) academic institutions, (4) labour associations, and (5) investors. These five main system-based actors are broadly discussed below. However, this is not an exhaustive list.

First, the government was noted to be an important system-based actor, as remarked by one interviewee from the Montreal region: “government is a key player in resilience. And that’s as an organization that can marshal resources, lead, and bring together both public and private sector to solve problems” (P32). Indeed, interviewees highlighted that the government, at various levels, support firm-based actors’ response to shocks through different programmes and policies (e.g. business incubators and accelerators, funding programmes, incentives, and loans, and so on). For example, one interviewee (P05) from the Toronto region noted that during the COVID-19 pandemic:

We built a program called [Digital Main St.] ShopHere, where we build online stores for Main Street retailers at no cost. So, we ramped up a program with Shopify. We recruited about 700 web developers from the tech community, and we built online stores for free. But we built that in like 10 days and launched it and ran. It ended up turning into a national program. As of now, they’ve built over 50,000 online stores. But again, it was just like, well, stores can’t open because of lockdown measures. People still need stuff. How do we connect people and make this happen?^4^

The previous example was just one of many referenced by interviewees. Overall, interviewees highlighted that the government plays a supportive role, to varying degrees, during shocks.

Second, business associations (e.g. local, provincial, and national Chambers of Commerce, Boards of Trade, Business Improvement Areas, and so on) were also noted to be key system-based actors. Specifically, business associations were highlighted to support firm-based actors to adapt during shocks, both directly through establishing various programmes and indirectly through advocating on behalf of their members to various levels of government. Concerning their advocacy role, one interviewee (P11) from the Ottawa-Gatineau region stated:

I would also add Chambers of Commerce, Boards of Trade and Business Improvement Areas to that list of key actors that support resilience. And I say that particularly from a lens of advocacy. It’s far easier for those external organizations . . . they are privy to having the Ontario Chamber of Commerce, the Canadian Chamber of Commerce, and that strength in numbers when there’s a collective issue happening across the business community. They may not be able to implement the programs that provide that sort of support or help businesses to weather shocks and get the businesses to a sustainable standpoint, but they’re at the precipice of the development of the programs because they’re informing and advocating in large numbers.^5^

Providing an illustrative example of business associations establishing programmes, one interviewee (P01) from the Toronto region commented:

The Chambers [of Commerce] and Board [of Trade], they have an impact. They make a big difference. Their members look to them for a voice and policy and advocacy and so on. The Chambers in Toronto worked together to run a series of workshops during the pandemic that supported businesses adapt . . .. And then another one, later on in the pandemic, they took the helm and said, okay, we’re going to do something, an accelerate business recovery hub.

Third, academic institutions were noted to support firm-based actors in responding to shocks. In particular, academic institutions were highlighted by interviewees to assist business start-ups and help businesses with labour shortages grow during shocks. With respect to the former, one interviewee from the Toronto region commented, “York University . . . since 2019 worked with almost 800 business start-ups. Seneca College . . . they served about 150 ventures. So, between Seneca and York, their tech hubs, you’re looking at nearly 1000 businesses and entrepreneurs served there” (P04). Concerning the latter, one interviewee from the Edmonton region answered, when asked who are key actors in regional economic resilience, “Obviously, post-secondary institutions are another primary one . . . as they are able to shift employee skill sets to industries that aren’t suffering, whether that be through certification programs or micro-credentialing or things like that” (P39).

Fourth, labour associations and agencies were highlighted to be important system-based actors that assisted firm-based actors in adapting during shocks. The role of labour associations and agencies was tied exclusively to job matching to meet the labour needs of businesses, as reflected by one interviewee from the Montreal region who noted, “So, there’s a number of key agencies that assist regions’ [economic] resilience. Employment agencies obviously are key actors in assisting industry find workforce and talent” (P34). The previous quote echoed other interviewees who emphasized the important role labour associations and agencies played in connecting (skilled) labour to businesses that want to grow and expand during crises.

Fifth, investors of various kinds (e.g. angel investors and venture capitalists) were highlighted by interviewees to be crucial system-based actors, who support firm-based actors to adapt in turbulent times. For example, when asked what actors influence regional economic resilience, one interviewee from the Ottawa-Gatineau region remarked, “Angel networks. They’re helpful as well. Very, very helpful” (P15). Expanding on this point, an interviewee from the Calgary region stated, “the guys that have the money, that are willing to invest it, the investors are the ones that, these are angel investors or venture capitalists or whoever. They see opportunity when things go down and they make it an opportunity for them” (P16). The previous quote reflected other interviewees’ comments that investors support businesses and entrepreneurs, especially innovative ones, during crises.

Interviewees constantly stressed that the “out-of-crisis” actions and behaviours of system-based actors also influence the economic resilience of regions. For instance, system-based actors were noted to aid the development of structural factors by working with firm-based actors, either by supporting business start-ups, helping local businesses expand, or attracting foreign businesses, and by helping preserve and increase regional enabling structural factors. Although the role that system-based actors play pre-crisis is not discussed in this paper, it is important to note to fully illustrate the complexities and dynamics underpinning the interaction between the determinants of resilience.

### Structural factors

Various structural factors were noted to influence regional economic resilience, such as industrial structure, sectoral composition, supply chain, and business characteristics. Since these noted structural factors have been discussed in the resilience literature (e.g. Martin and Sunley, 2015), only industrial structure will be elaborated on to illustrate how such factors, due to their structural nature, influence the economic resilience of regions.

The industrial structure of economies was the structural factor most cited by interviewees. They emphasized that a diversified industrial base enhances regional economic resilience, whereas a specialized one hinders it. As noted by one interviewee from the Ottawa-Gatineau region, “a diversified economy is going to be a resilient one” (P14). Or, as highlighted by an interviewee from the Montreal region, the problem with a specialized industrial base is “having all of your eggs in the same basket. So having a mono-industry. That’s a situation of vulnerability” (P33). Generally, interviewees discussed a diversified industrial structure in the context of either being comprised of a related or unrelated variety and indicated that different types of variety have different effects on the economic resilience of regions.

Related variety was noted to have an absorptive effect on the economic resilience of regions. Specifically, interviewees emphasized that the related variety of industries in similar sectors increased the ability of regions to absorb those laid off during crises due to the skilled relatedness of workers. Reflecting on this point, an interviewee (P37) from the Edmonton region commented:

So, yes, we’re industry heavy, but it’s not just oil, it’s not just natural gas, it’s not just hydrogen, it’s not just specialty metals. We have them all, which means that there’s a stability there for our workforce and for the people who are living here . . .. We are lucky because there’s so many shared labor profiles between all of the industries. So, even if one is kind of slowing down a bit, some of the others might be picking up and so that allows our workforce to transition kind of between those big industries. So, again, it’s a safety and kind of resilience there as well.

As suggested by the previous quote, interviewees emphasized the need for industries to be skill-related to enable workers to transition between industries for the absorptive effect to take hold. The only exceptions to this requirement indicated by interviewees were the downward transition of skilled workers to unskilled jobs or the horizontal transition of workers from one unskilled position to another, but in a different sector.

Unrelated variety was noted to enhance the economic resilience of regions through a portfolio effect. Specifically, interviewees highlighted that the unrelated variety of industries in different sectors acted as a risk-spreading strategy. This point was succinctly expressed by one interviewee from the Halifax region who commented, “the basic mathematical idea is that if your economy is spread across 20 different industries and one of them goes down, 19 of them are still up and running. And if your economy is based on four sectors, one of them goes down, well, then you just lost 25%” (P41). The previous quote echoes other interviewees who highlight that unrelated variety reduces the spread of shocks across sectors.

Furthermore, a diversified industrial base (of either type of variety) was emphasized to improve the economic resilience of regions through a recombinant effect, or, in other words, by enhancing the adaptive capacity of firm-based actors. Precisely, interviewees indicated that industrial diversity, compared to specialization, provides greater opportunities for firm-based actors from different industries and sectors to interact and recombine knowledge in novel ways, thereby generating innovation and boosting the economic resilience of regions. Illustrating this point, one interviewee (P02) from the Toronto region stated:

And so that diversity of different sectors is a key piece of resilience . . . there’s benefits in that amalgamation and that diversity and the creativity that can come from all of those. So, yes, you’ve got lots of manufacturing, but you’ve also got a strong tech community that probably means you can better access tech talent and AI talent to look at how you modernize your platform, your manufacturing processes or whatever, right? So, that diversity piece is another key part of resilience as well.

Or, as simply noted by an interviewee from the Ottawa-Gatineau region, highlighting the importance of industrial diversity, “just having that ability to have various different industries that work together and collaborate . . .. I would say a lot of it has to do with resilience and just being able to adapt to changes” (P12). Indeed, as reflected by the previous quotes and echoed by many interviewees, the diversity of industries and sectors enhances the potential for adaptive behaviour among firm-based actors during shocks.

### Enabling structural factors

Notably, interviewees emphasized that three main enabling structural factors enhance regional economic resilience: land availability, infrastructural capacity, and labour availability and diversity. In particular, these enabling structural factors were noted to be important as they enable firm-based actors to (1) develop the structural factors that boost regional economic resilience and (2) adapt to shocks.

Land availability (i.e. employment and residential land) was noted to be an important enabling structural factor. Interviewees, from all seven regions, stressed that land is not infinite and regions are limited in their ability to grow and expand. This point is expressed by one interviewee from the Montreal region who remarked, “a region is limited by its geographical limits” (P30). Or, as an interviewee from the Toronto region highlighted, “we don’t have vacant land anymore . . .. As Toronto begins to approach the reality, the realization, that land isn’t infinite, better start thinking about how to manage this . . . every region gets there in their own time” (P03). As a result, interviewees highlighted that the scarcity of vacant land could reduce the capacity of regions to develop the necessary structural factors, such as a diversified industrial base. For example, one interviewee from the Vancouver region commented, “Vancouver has been extremely vocal for a number of years about the need to intensify industrial land and find more industrial land. So, having the right kinds of land use planning to enable the diversity of businesses” (P26).

Furthermore, interviewees noted that vacant employment land enhances the adaptive capacity of regions during shocks. Specifically, vacant employment land provided firm-based actors the opportunity to expand or relocate their operations in or to a region. As expressed by one interviewee from the Toronto region, noting how their economy adapted during the COVID-19 pandemic, “we’re now close to 0% vacancy rate for industrial space. During the pandemic, with the shifting supply chains, some local businesses re-shored some production, which created new economic opportunities from a manufacturing perspective” (P08). The previous quote was echoed by many interviewees, highlighting the need for vacant employment land in order for the adaptive behaviour of firm-based actors to unfold during crises.

Interestingly, infrastructural capacity was also considered a crucial enabling structural factor. Specifically, infrastructure, such as electricity, internet connectivity, water, transportation, and services, among others, was highlighted to support the development of structural factors. Although in some cases, companies can develop the required infrastructure, having the necessary infrastructure can help attract businesses or enable local businesses to expand and hence, develop important structural factors. Expressing this point, one interviewee (P36) from the Edmonton region noted:

So, for instance, [a new company] is bringing billions in investment to Edmonton . . . because they’re able to do that here . . . there’s so many industrial companies, all within one tiny little area, we have a tremendous amount of existing infrastructure for them to take advantage of. So, it’s a little bit of plug and play. It’s a much less expensive investment than if they were to go someplace else that doesn’t already have that infrastructure. We have the rail, we have the pipelines, we have all of that.

Without the required infrastructure, regions can face difficulty developing important structural factors. As expressed by one interviewee (P20) from the Calgary region, who stated:

we’ve had a water shortage for quite a few years, and so now we’ve got funding for a water plant, which is changing a lot of things for us. And that’s why we never really grew as big as other cities, we just didn’t have the infrastructure to grow . . . right now, like even our businesses that are high [water] usage, we have to say no to them expanding. You really need to have that economic development infrastructure . . . it’s definitely having the infrastructure in place in order to allow businesses to grow, expand, and invest.

Infrastructural capacity was also noted to enhance the economic resilience of regions during turbulent times, not just pre-crisis, through supporting the development of structural factors. Specifically, interviewees indicated that having the necessary infrastructure supports the adaptive behaviour of firm-based actors during shocks. For instance, one interviewee from the Toronto region, discussing how firms in their region were able to adapt to the COVID-19 pandemic, commented, “the sort of shift to working from home wasn’t a big challenge for the companies in Toronto. Why? Because we have the digital infrastructure needed for that” (P05).

Labour availability and diversity were emphasized to be important enabling structural factors. Precisely, they allow businesses to grow, diversify, and operate. Concerning the importance of labour availability, this point was highlighted by one interviewee from the Montreal region who commented, “we have a lot of businesses that are struggling with labour shortages. They’re becoming vulnerable because they can’t grow their business, sometimes they can’t even complete their operations” (P32). Or, as an interviewee from the Vancouver region remarked, when asked what factors support regional diversification, “access to a healthy labour pool. So, making sure that if you have different types of businesses, that they can access the employees that they need. And also, healthy businesses require certain foundations and stability. And that comes from having a labour force that can grow with them” (P27). Emphasizing the importance of labour diversity, an interviewee from the Ottawa-Gatineau region answered, when asked what factors support the economic resilience of regions, said, “diversification would be a big one, just having various different sectors . . . and having the workforce to sustain those businesses as well . . .. So, that diversification, both on the workforce side, but also on the economic sector side would be key” (P11).

Labour availability and diversity were also noted to enhance firm-based actors’ response to shocks. Focusing on the former, one interviewee from the Edmonton region stated, “having a labour surplus has a lot to do with resilience and businesses just being able to adapt to challenges” (P35). Expanding on this point, interviewees noted that labour availability was typically needed to fully take advantage of opportunities that arose during crises, as remarked by one interviewee from the Vancouver region who stated, “during COVID, it was interesting to see the tourism side completely shut down, but then the logistics side just boomed. We still ended up having a worker shortage, because the logistics and industrial sector kind of snapped up all available workers” (P24). With respect to labour diversity, one interviewee (P16) from the Calgary region highlighted:

if you don’t have diverse skills across your workforce, you’re not going to be as quick to adapt to the local market conditions. So, the training and upgrading of skills takes three to five, six, seven years, depending on the trade or the area of expertise. Whereas, the speed of business moves overnight. So, having a diversity of skills is how we can be prepared overnight for tomorrow’s workforce . . . to be resilient, to be adaptable, to be open to those market conditions and changing conditions.

Overall, interviewees indicated that collectively the three main enabling structural factors support the development of structural factors and aid firm-based actors in responding to shocks. For instance, available land, infrastructure capacity, and labour availability and diversity are all needed for regions to develop a diversified industrial structure or for firm-based actors to expand or grow their operations during crises. Also, interviewees stated that the enabling structural factors are also crucial for attracting businesses, which support the recovery of regions following a shock. This latter point is not elaborated on as it closely reflects the discussion on how the enabling structural factors support the development of structural factors. However, this point is highlighted as it helps to fully illustrate the complexities and dynamics underpinning the interaction between the determinants of resilience.

### Interactions between the determinants

**Figure 2.**
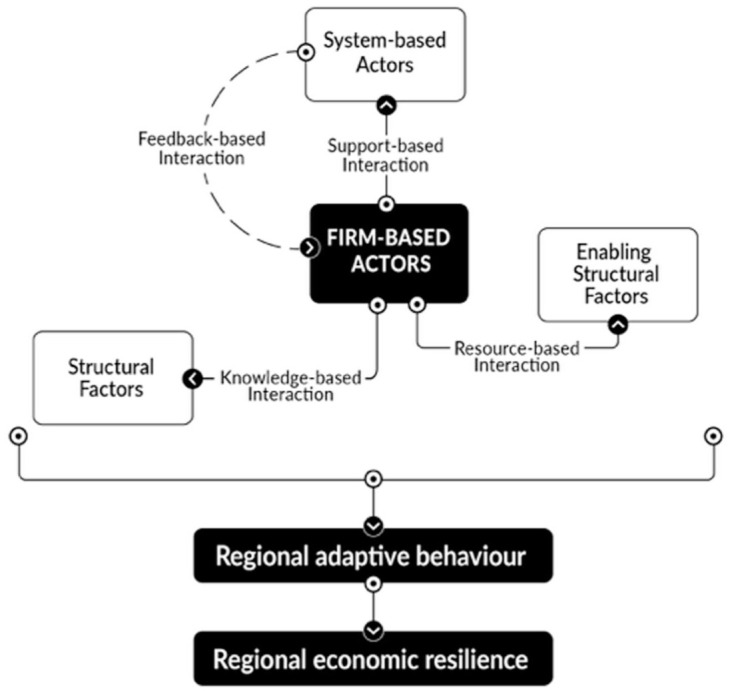
The interactive nature of the determinants of regional economic resilience.

As indicated above, firm-based actors are at the centre of each interaction (Figure 2). These interactions shape firm-based actors’ capacity to adapt to shocks, and the collective behaviour of regionally operating firm-based actors determines how regions adapt, thereby influencing their economic resilience. Firm-based actors engage with the other determinants of regional economic resilience (i.e. system-based actors, structural factors, and enabling structural factors), but in distinct ways. Specifically, firm-based actors interact with system-based actors by leveraging their support (e.g. information and financial resources) to adapt during crises. This type of interaction is largely support-based. Also, firm-based actors interact with structural factors by leveraging regional knowledge that is embedded in their economic and industrial fabric to adapt during turbulent times. This type of interaction is primarily knowledge-based. Lastly, firm-based actors interact with enabling structural factors by utilizing available regional infrastructure, labour, and land when adapting to shocks. This type of interaction is mainly resource-based. Regardless of the type that occurs, interactions influence the adaptive capacity of firm-based actors, which in turn shape the adaptive capacity and economic resilience of their region.

Notably, the discussion thus far indicates that interactions are linear. The appearance of a linear interactive relationship is because (1) interactions stem from actors who have agency and (2) interviewees primarily focused on how firm-based actors’ adaptive capacity during shocks is shaped by the other determinants.^6^ However, interviewees also implicitly noted that the relationship between firm-based and system-based actors during turbulent and stable times was circular and mutually reinforcing. For example, one interviewee (P05) from the Toronto region stated, “We reached out to our business community early on during COVID, held regular roundtables, and used what we heard to tweak our support programs. For example, after hearing that many small manufacturers couldn’t access PPE, we partnered with local firms to source and distribute it directly.” Or as another interviewee (P23) from the Vancouver region commented, “The Chamber of Commerce kept hearing from firms about the lack of skilled trades, so we worked with the province to expand apprenticeship seats. That change came directly from what businesses were telling us during COVID.” System-based actors’ interactions with firm-based actors are generally feedback-based. As indicated by the previous quote and echoed by other interviewees, feedback-based interactions can result in system-based actors altering or improving regional environmental factors, which were implied to occur during stable times as such changes take years to unfold.

## Discussion and conclusion

This paper provides an empirically grounded conceptual framework of the determinants of resilience. In doing so, it expands our current conceptual and empirical understanding. The main contribution of this paper to the resilience literature is threefold. First, the conceptual framework moves past providing a list or a set of factors that influence regional economic resilience by illustrating precisely how the determinants of resilience (i.e. actors and environmental factors) interact in a complex and dynamic manner. As a result, the empirically grounded conceptual framework corroborates previous assertions made by scholars (Hu and Hassink, 2020; Martin and Sunley, 2015, 2020) that the determinants do not operate in isolation. However, the framework goes beyond broad statements by demonstrating how such interactions unfold and providing empirical backing. In this vein, our framework differs from others, such as Martin and Sunley (2015), that highlight that interactions occur, but do not explain how they do so or the types that ensue.

Second, it highlights that the determinants of resilience, and regional economic resilience in general, must be understood within the ecological limits or “realities” of regions. Specifically, the ecological limits of regions emphasize that regional growth is bounded due to, among others, limited employment and residential land, and thus, regional economic resilience should be conceptualized as such. In this respect, regional ecological limits naturally point to the need to incorporate economic sustainability into future conceptualizations of regional economic resilience. By accounting for the ecological limits of regions, scholars in the resilience literature can avoid the pitfall of abstract conceptual development that is detached from reality (Chlebna et al., 2024; Yeung, 2024). Further, the importance of accounting for ecological limits (i.e. the degree to which environmental factors can be developed) is backed by literature on land, infrastructure, and economic development (Capello, 2015; Krawchenko and Tomaney, 2023; Leigh and Blakely, 2017; Rietveld, 1989), stressing that the “provision of infrastructure . . ., the development of transport networks and other public services (such as health and education) provide the frameworks within which development occurs” (Krawchenko and Tomaney, 2023: 616). Or, simply put, enabling structural factors represent the foundation upon which development occurs.

Third, it answers recent calls by scholars (Hu and Hassink, 2020; Lemke et al., 2023; Sutton et al., 2023) for greater exploration of the determinants of resilience. Specifically, the framework uncovers the role that indirect or secondary determinants (i.e. system-based actors and enabling structural factors) play in regional economic resilience, which has largely been neglected in the resilience literature. In addition, the framework provides greater insight into the range of actors (e.g. labour associations, educational institutions, investors, and business associations) that influence the economic resilience of regions and how they do so, which scholars (Bristow and Healy, 2014, 2020; Kurikka and Grillitsch, 2021; Lemke et al., 2023) argue is central for understanding the economic resilience of regions. Notably, the findings surrounding the role of system-based actors in regional economic resilience contribute to ongoing discussions in the broader literature (Hassink et al., 2019) concerning the influence of non-firm actors in regional development. Overall, this paper contributes to the resilience literature by providing a novel and insightful conceptual framework that (1) outlines in detail the interactive nature of the determinants of resilience, (2) emphasizes the ecological limits of regions, and (3) stresses the key role of actors (i.e. both firm and non-firm actors), without ignoring important environmental factors.

The developed conceptual framework of the determinants of resilience situates perfectly within the current and broader evolutionary conceptual framework of the resilience process put forth by Martin and Sunley (2015), Sutton et al. (2023), and others. At the onset of a crisis, the pre-crisis actions and behaviours of regionally operating actors influence the *preparedness* of regions; the structural factors primarily influence the *vulnerability* and *resistance* of regions; and the collective adaptive behaviour of regionally operating actors, interacting with environmental factors, largely determines the *response* and *recoverability* of regions. In this vein, the developed conceptual framework builds on and expands the current conceptualization of the resilience process. Furthermore, the developed framework provides a deeper understanding of the recursive process of resilience, especially regarding the role of enabling structural factors. For instance, actors’ adaptive behaviour during crises can shape regional land availability for development post-crisis and for adaptive behaviour during future shocks. In this sense, the developed framework offers greater insight into the historical evolutionary process of resilience (Martin and Sunley, 2015).

Moreover, the developed framework also emphasizes the role of institutions, such as government, in aiding and constraining the adaptive behaviour of regionally operating actors. In doing so, the framework points to the need to incorporate a geographical political economy perspective. While evolutionary economic geography and geographical political economy are separate perspectives in the broader field of economic geography (Chu et al., 2023), recent efforts have tried to merge these two complementary perspectives (Essletzbichler et al., 2023; MacKinnon et al., 2019) to create a “Geographical Evolutionary Political Economy.” Such a perspective would advance our current understanding of the determinants of resilience and thus, regional economic resilience in general.

Since the objective of the paper was to develop an empirically grounded conceptual framework, an exhaustive list of the identified determinants of resilience is not provided. In other words, many determinants are not discussed in this paper, such as the role of global value chains (Jicha et al., 2025). However, the groundwork for understanding the determinants of resilience is provided, allowing scholars to simply include other determinants into the developed framework. Indeed, further conceptual and empirical advancements building on the framework are expected.

## Notes

1. Since the focus of this paper is on the determinants of resilience, a greater overview of the process of economic resilience is not given. For such an overview, please see Martin and Sunley (2015) or Sutton et al. (2023).

2. The effects stemming from diversity and its different varieties will be covered in more depth in the results section and thus will not be further discussed here.

3. EDPs were from various types of municipalities, such as cities, counties, and regional municipalities. The type of municipality that EDPs operate in does not influence the results, but simply reflects the various scales at which economic development is undertaken.

4. See the following link for the Digital Main St. ShopHere website: https://digitalmainstreet.ca/Shophere/

5. For an example of business associations’ advocacy, see the list of letters sent to the government by the Ontario Chamber of Commerce: https://occ.ca/covid19-advocacy/

6. This is not to say that structural and enabling structural factors cannot influence the other actors, but they do not interact with actors as they lack agency.
